# Comparing low volume versus intermediate volume bowel preparation and their impact on work and tolerability: an open-label, noninferiority randomized controlled trial

**DOI:** 10.1055/a-2695-0994

**Published:** 2025-10-20

**Authors:** Milou L.M. van Riswijk, Fleur A. Indemans, Kimberly Hawinkels, Ramon M. Schreuder, Leanne Wildeman, Adriaan C. I. T. L. Tan, Peter D. Siersema

**Affiliations:** 1 6034 Gastroenterology and Hepatology, Radboud Institute for Health Sciences, Radboud University Medical Center, Nijmegen, Netherlands; 2 72489 Gastroenterology and Hepatology, Maasziekenhuis Pantein, Boxmeer, Netherlands; 3 3168 Gastroenterology and Hepatology, Catharina Hospital, Eindhoven, Netherlands; 4 3168 Division of Gastroenterology and Hepatology, Catharina Hospital, Eindhoven, Netherlands; 5 6034 Faculty of Medicine, Radboud University Medical Center, Nijmegen, Netherlands; 6 6030 Gastroenterology and Hepatology, Canisius Wilhelmina Hospital, Nijmegen, Netherlands; 7 6993 Gastroenterology and Hepatology, Erasmus MC University Medical Center Rotterdam, Rotterdam, Netherlands

## Abstract

**Background:**

Bowel preparation is essential for colonoscopy but may deter patients. Although low volume laxatives are better tolerated, their impact on patient-reported outcomes remains unclear. We compared low and intermediate volume bowel preparation and assessed their impact on tolerability, health-related quality of life (HRQoL), and work.

**Methods:**

We conducted an open-label, noninferiority randomized trial in four Dutch hospitals. Patients were randomized to 2 L polyethylene glycol with ascorbate (2L PEG+Asc) or 1 L with added sodium sulfate (1L PEG+Asc). Before and after preparation, patients completed validated questionnaires on productivity, tolerability, and HRQoL. The primary outcome was the proportion of patients with adequate bowel preparation, aiming to demonstrate noninferiority of 1L PEG+Asc vs. 2L PEG+Asc (5% noninferiority margin). Secondary outcomes included willingness to repeat, with exploratory analysis of associated factors using multivariable logistic regression, change in HRQoL scores, tolerability, and work-related impact.

**Results:**

We included 467 patients (2L PEG+Asc, n = 229; 1L PEG+Asc, n = 238). 1L PEG+Asc was noninferior to 2L: adequate cleansing rate, 96.1% (95%CI 92.6% to 98.0%) vs. 96.4% (95%CI 93.0% to 98.3%; *P* = 0.84; Δ −0.4, 95%CI −4.0 to 3.3). More patients in the 1L PEG+Asc group were willing to repeat the preparation (59.9% vs. 48.3%; *P* = 0.04), with tolerability the most influential factor (odds ratios 0.05 and 0.22 for difficult or fair vs. good tolerability, adjusted for symptoms, satisfaction, and 1L/2L PEG+Asc). No clinically relevant changes in HRQoL were observed. Absenteeism and impaired work productivity occurred in 7.9% and 12.3%, respectively, with no between-group differences.

**Conclusions:**

Bowel preparation with 1L PEG+Asc is noninferior to 2L PEG+Asc and associated with higher willingness to repeat. Tolerability is fundamental for effective cleansing and reducing colonoscopy barriers.

## Abstract (de)

**Graphical abstract:**

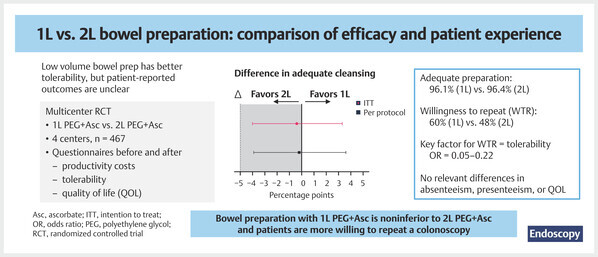

## Introduction

High quality colonoscopy cannot be performed without adequate bowel preparation 1 . Inadequate bowel preparation is associated with lower lesion detection rates and is a major predictor of failed cecal intubation. Moreover, the need for repeat procedures increases healthcare costs. Importantly, patients with poor bowel preparation are less likely to be satisfied about their colonoscopy and have a lower willingness to repeat colonoscopy 2 . This is detrimental to the benefit of colorectal cancer (CRC) screening, as lower participation rates significantly decrease colonoscopy screening efficiency 3 .

Efforts have been made to increase both bowel preparation efficiency and tolerability. Meta-analyses have demonstrated a higher tolerability of lower volume bowel preparation fluids 4 . A 2 L polyethylene glycol (PEG) solution with added ascorbate (2L PEG+Asc) has been shown to be noninferior to the “gold standard” of 4 L of PEG, and is associated with a higher willingness to repeat 1 . Recently, even lower volume bowel preparations have been introduced, such as 1L PEG+Asc with added sodium sulfate.

Nevertheless, the impact of bowel preparation on patients’ working lives and health-related quality of life (HRQoL) may be significant, potentially leading to postponement and sometimes to patients refraining from undergoing colonoscopy 2 5 . Despite its negative correlation with both bowel preparation efficacy and colonoscopy uptake, little is known about the effect of bowel preparation on patient-reported outcomes, and the differences between various volumes of laxative. In a prospective cohort of 1100 patients, Collatuzzo et al. reported a correlation between patient-related factors and symptoms caused by bowel preparation 6 . Fuccio et al. demonstrated that bowel preparation and colonoscopy significantly impacted work, with up to one-third of patients reporting work absenteeism or reduced performance, particularly when using same-day full-dose regimens or experiencing procedure-related symptoms 7 . They did not however include the 1L PEG+Asc regimen, and the studies were not randomized, which may lead to selection bias of patients choosing the laxative of their choice. Additionally, unemployed patients were excluded, while a large proportion of the patient population undergoing colonoscopy also consists of patients who are retired or not working owing to illness. Other studies on the impact of bowel preparation on patient-reported outcomes and work productivity are scarce and have mainly involved larger volume preparations (2 L and higher).

Insight into the impact on symptoms experienced, work, and HRQoL may help patients and caregivers in selecting a bowel preparation regimen and could reduce the barriers experienced to undergoing colonoscopy. Therefore, this study aimed to compare low volume and intermediate volume bowel preparation in terms of their bowel cleansing efficacy, tolerability, and impacts on work and HRQoL.

## Methods

### Study design

We performed an open-label, noninferiority randomized controlled trial in four Dutch hospitals. We included adult patients referred for diagnostic, screening, or surveillance colonoscopies. Exclusion criteria included: therapeutic procedures, inpatient status, emergency colonoscopy, inflammatory bowel disease, American Society of Anesthesiologists (ASA) score ≥4, (partial) colectomy, inability to provide informed consent, inability to complete Dutch questionnaires via email (owing to language barrier, lack of email, visual impairment, or dementia), common contraindications for bowel preparation or its ingredients, and indications for intensified bowel preparation as assessed by the treating physician.

The study was approved by the Radboud University Medical Center Medical Ethics Committee (Medisch Ethische Toetsingscommissie Oost-Nederland, registration number NL79014.091). It was conducted in accordance with the Declaration of Helsinki and Good Clinical Practice guidelines. All patients provided written informed consent. An independent monitor reviewed the study data and informed consent process, and two research team members independently verified all study data.

### Randomization

After informed consent had been obtained, eligible patients were randomized 1 : 1 using a block design with variable block sizes (4, 6, 8) via a secure web-based system (Castor Electronic Data Capture, Amsterdam, The Netherlands). Randomization was stratified only by study site and prior experience of bowel preparation. The randomization sequence was blinded to study team members, but patients and healthcare providers were not blinded to the allocation to minimize interference with routine medical care. Blinding of patients was not possible owing to the differing volumes. We included additional questions on patient experiences and satisfaction in the questionnaires to assess the impact of potential modifying factors without disrupting standard care.

### Study procedures

#### Bowel preparation and colonoscopy

Patients received either 1L PEG+Asc (Pleinvue, Norgine) or 2L PEG+Asc (Moviprep, Norgine) 8 in an overnight split-dose regimen, each dose followed by 0.5 L clear liquids (additional fluids were allowed to reflect real-life conditions and improve adherence). The last dose needed to be completed 2 hours before travel to the hospital. Patients were also instructed to follow a low residue diet starting 2 days before colonoscopy, followed by a liquid diet upon starting to take the laxatives. This was based on local clinical practice, although this does deviate from the European Society of Gastrointestinal Endoscopy (ESGE) bowel preparation guideline, which advises 1 day of low residue diet 1 . Instructions were provided by specialist nurses, supplemented with a detailed leaflet and patient diary to record the volume of additional fluids consumed.

Colonoscopies were performed as part of routine medical care, with quality control ensured under the Dutch national CRC screening program 8 , by experienced endoscopists, with the patients under conscious sedation or propofol. Bowel preparation quality was assessed using the Boston Bowel Preparation Scale (BBPS) after flushing, in line with routine clinical practice, with adequate preparation defined as a minimum score of 2 per segment 1 . High quality bowel preparation was defined as a BBPS of 3–3-3 (maximum score).

#### Questionnaires

Participants completed online questionnaires at two timepoints: before starting preparation (baseline) and within 1 week after colonoscopy. The first questionnaire collected baseline sociodemographic and lifestyle characteristics, clinical details including bowel symptoms, prior bowel preparation experience, risk factors for inadequate preparation based on the existing literature 9 , and HRQoL data. The second questionnaire assessed patients’ experiences with the bowel preparation and colonoscopy, impact on work productivity and costs, and HRQoL.

To assess work productivity impact, we used the validated Institute for Medical Technology Assessment Productivity Cost Questionnaire (IPCQ). Unlike the commonly used Work Productivity and Activity Impairment questionnaire, the IPCQ also considers the impact of unpaid labor 10 . HRQoL was assessed using the EuroQol Group five dimensions five levels (EQ-5D-5L). Previous studies have indicated that the endoscopic procedure minimally affects HRQoL outcomes, with bowel preparation being the primary factor influencing HRQoL 11 12 .

The second questionnaire also included the Mayo Florida Bowel Preparation Tolerability Questionnaire (MBTQ), validated in outpatient colonoscopy settings in the USA 13 . For our study, the MBTQ was translated using a forward–backward translation process by a professional translator. In addition to the MBTQ, we assessed patient satisfaction using the Dutch Gastrointestinal Endoscopy Satisfaction Questionnaire (D-GESQ) 14 . Finally, we recorded self-reported post-procedural adverse events (AEs) with the second questionnaire, and supplemented this with information from hospital records.

### Outcomes and objectives

Our primary outcome was the proportion of patients with adequate bowel preparation, defined as a segmental BBPS score of ≥2. The aim was to demonstrate noninferiority of 1L PEG+Asc vs. 2L PEG+Asc. Secondary outcomes included tolerability (assessed via the MBTQ), HRQoL (measured by EQ-5D-5L), and work productivity (evaluated using the IPCQ) to provide an exploratory comprehensive assessment of patient experience before and after bowel preparation.

### Statistical analysis

Baseline characteristics were summarized using mean and SD, median and interquartile range (IQR), or proportions, as appropriate. Comparisons were made using Student’s *t* test, Mann–Whitney *U* test, Wilcoxon’s rank test, chi-squared test, or Fisher’s exact test.

Primary outcome analysis was conducted on both an intention-to-treat (ITT) and per-protocol basis. The difference between the two groups is presented with a 95%CI. The noninferiority margin was set to 5%. If noninferiority was confirmed, superiority was tested using Fisher’s exact test. Questionnaire outcomes were analyzed per their manuals. The EQ-index of the EQ-5D-5L was compared with the general population 15 . A minimal clinically important change was defined as 0.5 SD of the change 16 . Costs of absenteeism, presenteeism, and unpaid labor were calculated using Dutch government-set reference costs (Zorginstituut Nederland), with hourly costs of €34.75 for paid work and €14 for unpaid labor.

Although this study was a randomized controlled trial and not designed for prediction modeling, we performed exploratory multivariable logistic regression to identify factors potentially associated with willingness to repeat the bowel preparation regimen. These analyses were conducted to better understand patient-reported experiences, and not for causal inference or predictive accuracy. The intervention group was included as a covariate in the model, and other variables were selected based on the literature and outcome events. After linearity and multicollinearity had been assessed, variables with a *P* value <0.2 on univariable analysis were included in the multivariable analysis, using backward propagation for model selection. Odds ratios (ORs) and 95%CIs were reported. Missing questionnaire data were handled through multiple imputation after confirming data were missing at random, using predictive mean matching with 50 iterations to create 50 imputed datasets.

All analyses were performed using SPSS statistics (IBM, version 25.0), with a two-sided significance level of 5%. Secondary outcomes were assessed in an exploratory fashion, therefore multiple-testing corrections were not applied.

### Sample size calculation

Assuming a 95% adequate preparation rate in both groups and a noninferiority margin of 5%, a sample of 470 patients (235 per group) was calculated as being required to demonstrate noninferiority using one-sided testing, with an alpha of 5% and 80% power.

## Results

### Baseline characteristics

**Fig. 1:**
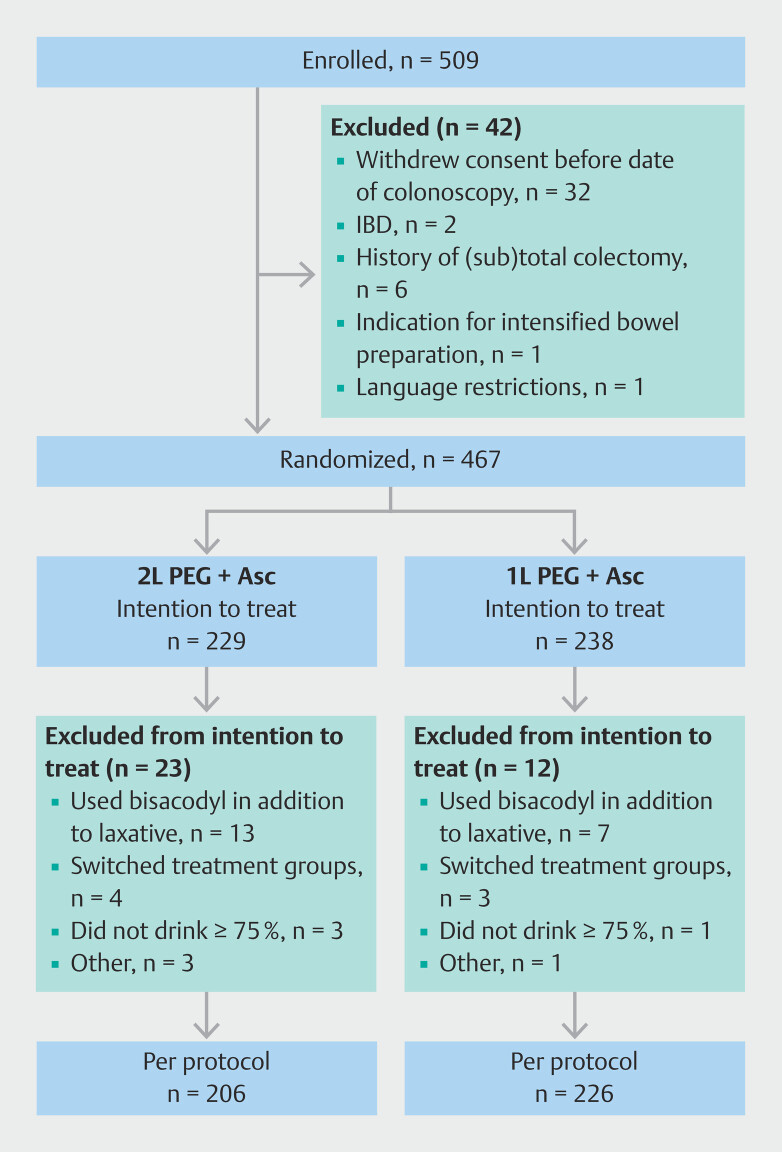
Flow chart of patient inclusions and exclusions. IBD, inflammatory bowel disease; PEG+Asc, polyethylene glycol with added ascorbate.

From May 2022 to February 2023, we enrolled 509 patients across four centers. After 42 patients had been excluded because of consent withdrawal or other exclusion criteria, 238 were randomized to the 1L PEG+Asc group and 229 to the 2L PEG+Asc group. The ITT population included 467 patients. In the per-protocol analysis, 35 patients were excluded owing to noncompliance or logistical treatment arm switches, resulting in 432 patients ( Fig. 1 ).

The baseline characteristics of the two groups were well balanced ( Table 1 ). The questionnaire response rate was 87.8% (n = 410) and was not significantly different between groups ( *P* = 0.32). Half of the study population (49.3%) had previous experience with bowel preparation, with 2L PEG+Asc being the most common prior regimen (24.9% and 29.8%). As for pre-existent risk factors for inadequate bowel preparation, most patients had a fair physical activity level, and 88.2% and 89.9% did not have any risk factors for poor bowel preparation. The background of the patients was predominantly Dutch (96.2% and 96.6%), with intermediate to high levels of education; around half were employed (51.7% and 54.0%).

**Table:** **Table 1** Baseline characteristics of the patients in the two groups that received either 1 L or 2 L of polyethylene glycol (PEG) and ascorbate (Asc) in the intention-to-treat (ITT) analysis.

	1L PEG+Asc (n = 238) ^1^	2L PEG+Asc (n = 229) ^2^
Response rate, n (%)		
Baseline	223 (93.7)	206 (90.0)
After colonoscopy	218 (91.6)	204 (89.1)
Both questionnaires	212 (89.1)	198 (86.5)
Median time between questionnaires in days (IQR)	8 (4–13)	9 (5–14)
Age, median (IQR), years	63 (55–71)	64 (55–70)
Sex, female, n (%)	101 (42.6)	98 (42.8)
ASA score, median (IQR)	2 (1–2)	2 (1–2)
BMI, median (IQR)	25.9 (23.6–29.7)	26.3 (23.4–29.4)
Prior experience with bowel preparation, n (%)	113 (49.3)	113 (47.5)
Number of prior colonoscopies, median (IQR)	2 (1–3)	2 (1–3)
Prior laxative used, n (%)		
2L PEG+Asc	71 (29.8)	57 (24.9)
3L PEG+Asc or more	7 (2.9)	14 (6.1)
1L PEG+Asc	3 (1.3)	3 (1.3)
300mL sodium picosulfate magnesium citrate	2 (0.8)	4 (1.7)
4L PEG	9 (3.8)	7 (3.1)
Other	3 (1.3)	2 (0.9)
Do not remember	34 (14.3)	34 (14.8)
Willingness-to-repeat prior laxative, n (%)		
2L PEG+Asc	65 (91.5)	49 (86.0)
3L PEG+Asc or more	6 (85.7)	13 (92.9)
1L PEG+Asc	3 (100.0)	3 (100.0)
300mL sodium picosulfate magnesium citrate	2 (100.0)	4 (100.0)
4L PEG	7 (77.8)	6 (85.7)
Other	2 (66.7)	2 (100.0)
Do not remember	28 (82.4)	26 (76.5)
Colonoscopy indication, n (%)		
Screening	75 (31.5)	89 (38.9)
Surveillance	77 (32.4)	71 (31.9)
Diagnostic	84 (35.3)	
Smoking, n (%)		
Active smoker	26 (11.1)	25 (11.2)
Former smoker	113 (48.3)	107 (48.0)
Never	94 (40.2)	91 (40.8)
Migration background, n (%)		
Dutch	217 (96.9)	200 (96.2)
Western migration background	3 (1.3)	5 (2.4)
Non-Western migration background	2 (0.9)	0
Other	0	2 (0.9)
Prefer not to say	2 (0.9)	1 (0.5)
Education level, n (%)		
None	3 (1.3)	0
Primary school	4 (1.8)	3 (1.4)
Secondary school or vocational college	126 (58.0)	119 (57.5)
University or college	89 (39.8)	82 (39.6)
Other	2 (0.9)	3 (1.4)
Marital status, n (%)		
Single	21 (9.4)	20 (9.6)
Married	149 (66.5)	150 (72.1)
Partner, living together	28 (12.5)	25 (12.0)
Partner, not living together	10 (4.5)	4 (1.9)
Widow(er)	14 (6.3)	7 (3.4)
Other	2 (0.9)	2 (1.0)
Physical activity level, n (%)		
Bedridden	0	2 (1.0)
Sedentary work	9 (4.0)	3 (1.4)
Work involving walking, no heavy lifting	118 (52.7)	100 (48.3)
Work involving walking and heavy lifting	43 (19.2)	33 (15.9)
Particularly strenuous physical work	54 (24.1)	69 (33.3)
Paid job, n (%)		
Yes	121 (54.0)	107 (51.7)
No	103 (46.0)	100 (48.3)
Work activity per week, mean (SD)		
Hours	31.8 (11.7)	32.6 (11.4)
Days	4.2 (1.1)	4.3 (1.1)
Occupational level, n (%)		
Student	1 (0.4)	2 (1.0)
Employed	90 (40.2)	79 (38.2)
Entrepreneur	19 (8.5)	19 (9.2)
Homemaker	17 (7.6)	6 (2.9)
Unemployed	2 (0.9)	1 (0.5)
Incapacitated for work	7 (3.1)	7 (3.4)
Retired	82 (36.6)	85 (41.1)
Other	6 (2.7)	8 (3.9)
Risk factors for poor bowel preparation, n (%)		
Related conditions		
None	214 (89.9)	202 (88.2)
Constipation	28 (11.8)	26 (11.4)
Abdominal surgery	27 (11.3)	27 (11.8)
Diabetes mellitus	22 (9.2)	18 (7.9)
Kidney disease	3 (1.3)	1 (0.4)
Liver cirrhosis	3 (1.3)	0 (0)
Parkinson’s disease	1 (0.4)	1 (0.4)
CVA	4 (1.7)	4 (1.7)
Medication		
Polypharmacy	191 (80.3)	189 (79.9)
Opioid use	6 (2.5)	3 (1.3)
TCA use	3 (1.2)	2 (0.9)
Bristol stool scale score, n (%)		
1	5 (2.2)	4 (1.9)
2	14 (6.3)	8 (3.9)
3	39 (17.4)	39 (18.8)
4	91 (40.6)	87 (42.0)
5	31 (13.8)	29 (14.0)
6	39 (17.4)	36 (17.4)
7	5 (2.2)	4 (1.9)
Median (IQR)	4 (3–5)	4 (4–5)
History of inadequate bowel preparation, n (%)	7 (6.5)	6 (6.1)
ASA, American Society of Anesthesiologists; BMI, body mass index; CVA, cerebrovascular accident; IQR, interquartile range; TCA, tricyclic antidepressant. ^1^ Missing data, n = 14 (5.9%). ^2^ Missing data, n = 22 (9.6%).		

### Bowel cleansing efficacy

**Fig. 2:**
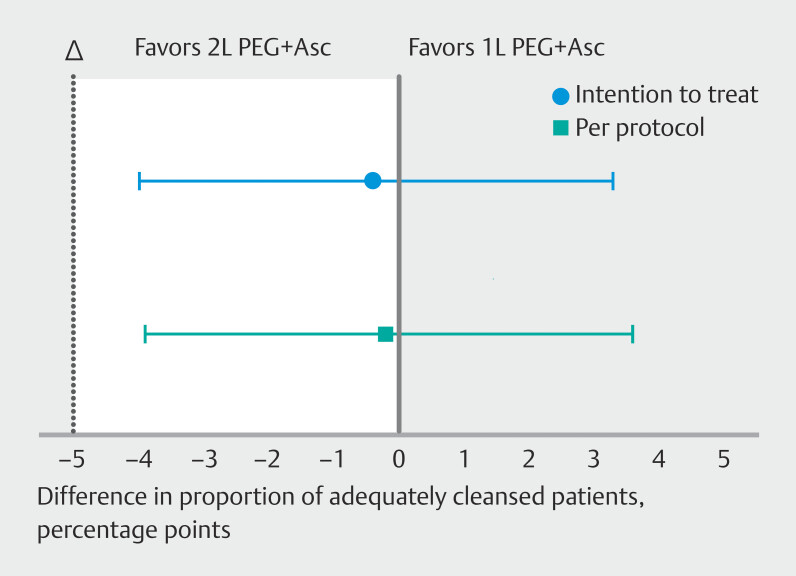
Proportion of adequately cleansed patients using 1L or 2L polyethylene glycol (PEG) bowel preparation (95%CI). The difference falls within our predefined noninferiority margin of 5% (∆), establishing noninferiority.

In the 1L PEG+Asc group, 96.1% (95%CI 92.6% to 98.0%) and 96.4% (95%CI 92.9% to 98.3%) of colonoscopies were adequately prepared in the ITT and per-protocol analyses, respectively, compared with 96.4% (95%CI 93.0% to 98.3%) and 96.6% (95%CI 92.9% to 98.5%), respectively, in the 2L PEG+Asc group (intention to treat, *P* = 0.84; per protocol, *P* = 0.92) ( Fig. 2 ; **Table 2** ). The between-group difference was −0.4 percentage points (95%CI −4.0 to 3.3 percentage points) for the ITT analysis and −0.2 percentage points (−3.9 to 3.6 percentage points) for the per-protocol analysis. This met our predefined 5% noninferiority margin.

The median BBPS scores were significantly higher in the 1L PEG+Asc group for the right ( *P* = 0.01) and transverse colon ( *P* = 0.004), but not significantly different in the left colon. High quality cleansing (BBPS 3–3-3) was more common in the 1L PEG+Asc group, with 72.7% (95%CI 67.0% to 78.0%) vs. 63.8% (95%CI 57.5% to 70.0%) in the 2L PEG+Asc group for the ITT population ( *P* = 0.04). Because of the high rates of adequate cleansing, in-depth analysis of the reasons of inadequate bowel preparation was not performed. Other colonoscopy quality parameters are detailed in Table 2 .

**Table:** **Table 2** Comparison of colonoscopy and bowel preparation outcomes for the patients in the two groups that received either 1 L or 2 L of polyethylene glycol (PEG) and ascorbate (Asc).

	1L PEG+Asc ^1^	2L PEG+Asc ^2^	*P* value
**Intention-to-treat analysis**	**n = 238**	**n = 229**	
Adequate cleansing ^3^ , n (%) [95%CI]	221 (96.1) ^4^ [92.6 to 98.0]	217 (96.4) ^5^ [93.0 to 98.3]	0.84
Difference in proportions, % (95%CI)	−0.4 (−4.0 to 3.3)		
High quality cleansing ^6^ , n (%) [95%CI]	173 (72.7) [67.0 to 78.0]	146 (63.8) [57.5 to 70.0]	**0.04**
**Per-protocol analysis**	**n = 220**	**n = 203**	
Adequate cleansing ^3^ , n (%) [95%CI]	212 (96.4) [92.9 to 98.3]	196 (96.6) [92.9 to 98.5]	0.92
Difference in proportions, % (95%CI)	−0.2 (−3.9 to 3.6)		
High quality cleansing ^6^ , n (%) [95%CI]	166 (73.5) [67.7 to 78.8	131 (63.6) [57.0 to 70.2	**0.03**
BBPS score, median (IQR)			
Right	3 (3–3)	3 (2–3)	**0.01**
Transverse	3 (3–3)	3 (3–3)	**0.004**
Left	3 (3–3)	3 (3–3)	0.08
Adequate cleansing stratified per center, n (%)	(P = 0.41)	(P = 0.34)	
Center a	36 (92.3)	32 (91.4)	
Center b	51 (98.1)	51 (96.2)	
Center c	55 (98.2)	55 (98.2)	
Center d	79 (95.2)	79 (97.5)	
Experience complying with low residue diet, n (%)			0.25
Very easy	50 (22.6)	56 (27.3)	
Easy	122 (55.2)	101 (49.3)	
Fair	37 (16.7)	42 (21.0)	
Difficult	11 (5.0)	5 (2.4)	
Very difficult	1 (0.5)	0 (0)	
Experience complying with liquid diet, n (%)			0.75
Very easy	39 (17.6)	40 (19.5)	
Easy	103 (46.6)	97 (47.3)	
Fair	58 (26.2)	48 (23.4)	
Difficult	16 (7.2)	18 (8.8)	
Very difficult	5 (2.3)	2 (1.0)	
Additional liquids taken, median (IQR), L	3 (2–4)	4 (2.4–5.1)	**<0.001**
≥75% compliance with laxative, n (%)	218 (99.5)	202 (98.5)	0.36
Physical activity level during bowel preparation, n (%)			0.81
Less than usual	85 (38.5)	85 (41.5)	
Not changed	131 (59.3)	116 (56.6)	
More than usual	5 (2.3)	4 (2.0)	
Cecal intubation, n (%)	219 (92.8)	219 (96.1)	0.13
Reason for failed cecal intubation, n (%)			0.10
Inadequate bowel preparation	3 (17.6)	0 (0)	
Obstruction/stricture	5 (29.4)	0 (0)	
Pain/discomfort	5 (29.4)	6 (66.7)	
Looping	4 (23.5)	3 (33.3)	
Gloucester comfort scale score, median (IQR)	2 (1–2)	2 (1–2)	0.60
Sedation, n (%)			0.18
No sedation	16 (6.8)	16 (7.0)	
Conscious sedation	207 (87.7)	207 (90.8)	
Deep sedation	13 (5.5)	5 (2.2)	
Adenoma detection rate, %	48.7	51.5	0.40
Adenomas per colonoscopy, median (IQR)	0 (0–1)	0 (0–1)	0.64
Serrated polyp detection rate, %	17.6	16.2	0.67
Colorectal cancer, n (%)	10 (4.2)	4 (1.8)	0.12
Polyp detection rate, %	55.9	62.0	0.18
Polyps per colonoscopy, median (IQR)	1 (0–2)	1 (0–2)	0.55
Withdrawal time, median (IQR), minutes	12 (8–17)	13 (9–19)	**0.047**
Asc, ascorbate; BBPS, Boston Bowel Preparation Scale; IQR, interquartile range; PEG, polyethylene glycol. ^1^ Missing from questionnaires, n = 17 (7.1%). ^2^ Missing from questionnaires, n = 24 (10.5%). ^3^ Adequate bowel preparation was defined as a minimal segmental BBPS score of ≥2. ^4^ Complete BBPS could not be assessed owing to incomplete colonoscopy, n = 8. ^5^ Complete BBPS could not be assessed owing to incomplete colonoscopy, n = 4. ^6^ High quality bowel preparation was defined as a BBPS score of 3 in all segments.			

### Impact on work and related costs

**Fig. 3:**
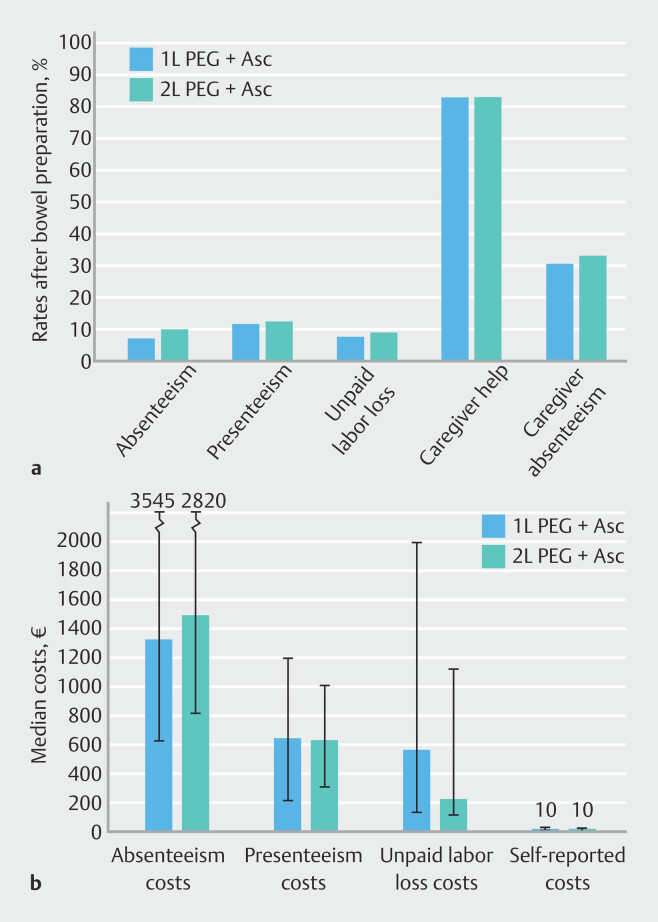
Bar charts comparing the 1L and 2L PEG+Asc groups for: **a** the impact on work and work productivity measured after bowel preparation and colonoscopy (rates of absenteeism and presenteeism calculated in the working subpopulation; rates of unpaid labor loss, caregiver help, and caregiver absenteeism in the entire study population); **b** the costs of absenteeism, presenteeism, unpaid labor loss, and median self-reported costs in euros.

No clinically relevant differences in the proportions of absenteeism or presenteeism were present between the groups ( Fig. 3 ; **Table 1s** , see online-only Supplementary material). Among working patients, the mean baseline absenteeism was 16.2%, decreasing to 7.9% after the colonoscopy. Additionally, 12.3% of patients in both groups reported reduced productivity (presenteeism) in the preceding 4 weeks, with median associated costs of €1390.

Unpaid labor loss rates remained stable before and after the procedure in both groups (9.0% before vs. 8.5% after colonoscopy), with mean costs of €1012 and €1065, respectively. Caregiver involvement was common, with 82.5% of patients requiring assistance, resulting in caregiver absenteeism in 30.6% of cases.

The out-of-pocket costs of patients were modest, with median out-of-pocket costs of €15 (IQR €8.5–49.5), and no significant difference between the study groups ( *P* = 0.59). No significant differences between the study arms were observed in any categories.

### Tolerability and impact on quality of life

#### Tolerability

Most respondents drank ≥75% of the bowel preparation fluid (99.5% in the 1L PEG+Asc group and 98.5% in the 2L PEG+Asc group; *P* = 0.18) ( **Table 2s** ). Tolerability was rated as “easy” by 51.6% in the 1L PEG+Asc group and 57.1% in the 2L PEG+Asc group ( *P* = 0.22). Willingness to repeat taking the bowel preparation was higher with 1L PEG+Asc (59.8% vs. 48.3%; *P* = 0.04). Among patients with previous experience of bowel preparation, more patients in the 1L PEG+Asc group found the tolerability better than their previous experience (26.9% vs. 9.3%; *P* < 0.001). The total symptom scores from bowel preparation were not significantly different between the groups, but more patients in the 1L PEG+Asc group reported a moderate or severe bad taste and nausea/vomiting compared with the 2L group ( **Table 2s** ).

On univariable regression analysis, married or living together (OR 1.86, 95%CI 1.12 to 3.09), higher endoscopy satisfaction scores (OR 1.07, 95%CI 1.04 to 1.10), higher score on the visual analog scale in the HRQoL questionnaire (EQ-VAS; OR 1.03, 95%CI 1.02 to 1.05), and EQ-index (OR 1.04, 95%CI 1.02 to 1.06) were significantly associated with a higher willingness to repeat the bowel preparation. In contrast, 2L PEG+Asc vs. 1L PEG+Asc (OR 0.62, 95%CI 0.43 to 0.90), female sex (OR 0.49, 95%CI 0.34 to 0.71), fair tolerability (OR 0.18, 95%CI 0.11 to 0.29) and difficult tolerability compared with good tolerability (OR 0.03, 95%CI 0.01 to 0.07) were significantly associated with a lower willingness to repeat the bowel preparation.

On multivariable analysis, lower tolerability (fair, OR 0.23, 95%CI 0.13 to 0.40; difficult, OR 0.05, 95%CI 0.02 to 0.14) and higher symptom score from bowel preparation (OR 0.85, 95%CI 0.78 to 0.93) had a negative impact on willingness to repeat the bowel preparation, while patients with a higher endoscopy satisfaction score (OR 1.04, 95%CI 1.01 to 1.08), and patients who received 1L PEG+Asc (OR 2.61, 95%CI 1.59 to 4.27)) compared with 2L PEG+Asc (OR 0.40) had a higher willingness to repeat the bowel preparation ( *P* < 0.001) ( **Table 3s** ).

#### Health-related quality of life

**Fig. 4:**
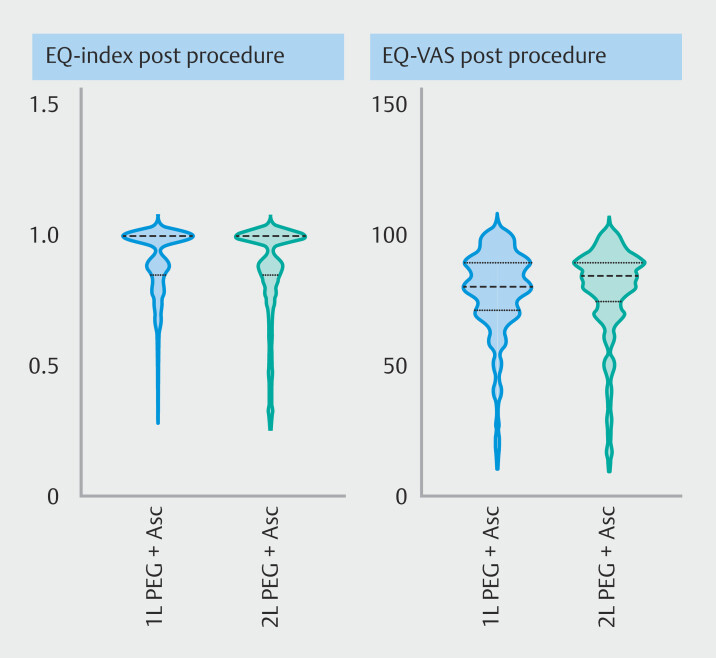
Violin plots of health-related quality of life outcomes measured by the EQ-index and EQ-VAS score in the 1L and 2L PEG+Asc groups. Higher scores indicate higher quality of life. A wider plot indicates more frequent occurrence of that score. Scores were not significantly different between the treatment arms.

In the EQ-5D-5L, patients scored a median baseline EQ-index of 0.89 (1L PEG+Asc) and 0.92 (2L PEG+Asc), both improving to 1.0 after colonoscopy ( *P* < 0.001). Both groups showed a slight decrease in EQ-VAS after the procedure, reflecting a modest drop below the minimum clinically important difference in perceived health status. The similarity in score change between the arms suggests that the choice of bowel preparation did not differentially impact overall perceived health ( **Table 4s** ). No differences were observed between the study arms in the subdomains ( Fig. 4 ; **Table 4s** ), although pain and anxiety in the 1L PEG+Asc group and mobility in the 2L PEG+Asc group significantly decreased below the minimum clinically important difference.

#### Endoscopy satisfaction

In general, patients were satisfied about the endoscopic care with a mean score of 85.1 (SD 8.3) ( **Table 5s** ). Patients were especially satisfied about the endoscopist’s skills (mean 92.4 [SD 8.8]) and overall hospital impression (mean 90.5 [SD 11.5]). Scores were not significantly different between the treatment groups.

### Adverse events

Sixteen AEs were reported, equally split between the 1L PEG+Asc and 2L PEG+Asc groups. In the 1L PEG+Asc group, all AEs were related to the colonoscopy. In the 2L PEG+Asc group, five AEs were colonoscopy related, while two involved bowel preparation (both atrial fibrillation) – these cases resolved within 1 day, but the colonoscopies were postponed and alternative laxatives were used. One serious AE, a head injury 30 days post-procedure, was unrelated to the colonoscopy or bowel preparation.

## Discussion

While the tolerability of bowel preparation is negatively associated with both bowel preparation efficacy and colonoscopy participation, little is known about the impact on patient-reported outcomes of bowel preparations with presumed higher tolerability owing to their lower volumes. Our findings demonstrate that the low volume preparation (1L PEG+Asc) was noninferior to the intermediate volume preparation (2L PEG+Asc) in achieving adequate bowel cleansing (96.1% vs. 96.4%), had higher high quality cleansing rates (72.7% vs. 63.8%), and was associated with greater patient willingness to repeat the preparation (59.8% vs. 48.3%). Patient-reported outcomes, including HRQoL and productivity loss, were not significantly different between the treatment arms.

Adequate efficacy is a prerequisite for research on patient-reported outcomes of bowel preparation. The noninferiority of 1L PEG+Asc to 2L PEG+Asc in our study is in line with previous studies 17 18 19 . Our rate of 96% adequately cleansed colonoscopies is at the high end of the spectrum. This could be due to the exclusion of patients at risk for inadequate bowel preparation from our study; however, this patient group has also been excluded from other studies 19 20 21 . The high compliance in both groups could also be a contributing factor. Furthermore, we included a low residue diet and split-dose protocol, combined with enhanced instructions, all known to improve efficacy and adherence 1 .

Our results demonstrated that tolerability is likely a critical factor in bowel preparation because it directly impacts patients’ compliance and their willingness to repeat, with patients who found the preparation “difficult” being 94.7% less likely (OR 0.05) to be willing to repeat the preparation compared with those who found it “easy.” Repici et al. demonstrated that 1L PEG+Asc had significantly higher compliance compared with 4L PEG, resulting in more effective bowel cleansing 19 . Similarly, Bednarska et al. concluded that patients were more willing to repeat a regimen of 1L PEG+Asc than 2L PEG+Asc 18 , consistent with our findings (59.8% vs. 48.3%; *P* = 0.04). Despite a more frequently reported bad taste ( *P* = 0.007) and nausea/vomiting ( *P* = 0.006) in the 1L PEG+Asc group, overall tolerability rates were similar between the groups. Patients who had prior experience with taking bowel preparation found the 1L PEG+Asc regimen significantly more tolerable than the bowel preparation they had taken previously, as also reported by Bednarska et al. 18 . This may well suggest that volume is an important factor in negatively affecting patient experience, even more than taste or side effects. Therefore, the superior experience of 1L PEG+Asc contributes to the efficacy of the bowel preparation. Furthermore, management of discomfort and anxiety by effective communication likely also plays an important role in improving compliance and overall patient experience 22 .

Because of the multiple instructions patients have to adhere to during bowel preparation, and possible discomfort and anxiety 2 , we hypothesized that these could also have an impact on HRQoL. We found comparable HRQoL scores to the general Dutch population, with no clinically relevant difference in either group. Andronis et al. reported that patients undergoing colonoscopy experienced a temporary decrease in HRQoL owing to bowel preparation discomfort 23 . In contrast, Niv et al. observed that, while patients had some discomfort during bowel preparation, the overall HRQoL scores remained relatively stable after colonoscopy, in line with our results 11 . As patients report some discomfort during bowel preparation, traditional HRQoL measures are unlikely to be able to fully capture the impact on patients 24 ; however, given the short duration of the bowel preparation process, any long-term impact on HRQoL by bowel preparation is likely negligible.

Bowel preparation can have a significant impact on patients’ work as they need to take time off to complete the bowel preparation and undergo the colonoscopy. Fuccio et al. reported in a prospective cohort (n = 1137) that absenteeism or presenteeism owing to bowel preparation or symptoms after colonoscopy was 30.5% in patients undergoing colonoscopy 7 . Our study’s lower rate of both absenteeism (7.9%) and presenteeism (12.3%) may be due to the use of a split-dose regimen and a difference in the laxatives used (4L and 2L vs. our study’s 1L and 2L). Other studies have observed absenteeism in 20%–32% of patients owing to bowel preparation, with an association found between a higher symptom burden and absenteeism 25 26 . Additionally, our study observed that the loss of unpaid labor was 8.5% after colonoscopy, which has been poorly investigated so far. Both direct (hospital) and indirect nonmedical costs are needed for a clearer understanding of the total expenses associated with bowel preparation 27 28 . Our findings may therefore offer a perspective on these costs from both patient and societal viewpoints.

Ongoing interest needs to be paid to bowel preparation tolerability to ensure adequate preparation but also to minimize the possible negative impact on patients. Possible variations include a split-dose regimen, diet liberations such as low residue diets with shorter durations 29 30 , and also developing other low volume laxatives. This will likely reduce the barriers experienced to colonoscopy and CRC screening 31 . Furthermore, our results on HRQoL and patient costs, using validated instruments, may inform healthcare policies and allow policies to be based on informed decisions that improve CRC screening participation, while minimizing impact on patients.

Our study has several strengths. In contrast to previous studies, our multicenter randomized design reduces the risk of bias. Additionally, we used validated instruments to increase generalizability and comparability. To minimize possible remaining bias, we used multiple imputations to compensate for missing information, although the response rate was already nearly 90%. The use of clinical and socioeconomic variables enabled us to correct for potential confounders and provided more optimal information on the impact of bowel preparation on patients and relatives.

We also acknowledge some limitations. The 2-day low residue diet was not in line with ESGE recommendations for bowel preparation and might potentially have influenced our results, but it is unlikely it would have introduced differences between the study groups. Additionally, as our secondary analyses were only performed exploratorily, external confirmation is needed. Our study sample lacked patients of non-Dutch ethnicity and those with lower education levels. This may limit generalizability to patients with other ethnicities or lower socioeconomic levels, while these patients are known to be at slightly higher risk for inadequate bowel preparation 9 32 . Furthermore, because of the mixed colonoscopy indications, the adenoma detection rate (ADR) presented in this study may limit comparison with fecal immunochemical test (FIT)-only cohorts, but the inclusion of various colonoscopy indications improves generalizability to a wider patient group. Lastly, although we are confident that relevant post-procedural AEs were captured through the questionnaires and hospital records, smaller events outside the hospital system may have been missed.

In conclusion, low volume 1L PEG+Asc bowel preparation is an effective and tolerable alternative to an intermediate volume 2L PEG+Asc preparation, with high patient satisfaction and minimal impact on quality of life and productivity. Prioritizing tolerability of bowel preparation is essential to increase adequate bowel cleansing and reduce barriers to colonoscopy.

## Supplementary Material

Supplementary Material Zusatzmaterial
