# Supplementary material for: Comparing low volume versus intermediate volume bowel preparation and their impact on work and tolerability: an open-label, noninferiority randomized controlled trial
Source: Endoscopy. 2025 Oct 20;58(2):151–62. doi: 10.1055/a-2695-0994 (PMC12828908; doi:10.1055/a-2695-0994)
Supplement: Supplementary file 1 — Supplementary Material [file 10-1055-a-2695-0994_27155941.pdf]

Supplementary material

Comparing low-volume vs. intermediate-volume bowel preparation and their impact on work and tolerability: an open-label noninferiority randomized controlled trial

Milou L.M. van Riswijk, Fleur A. Indemans, Kimberly Hawinkels, Ramon M. Schreuder, Leanne Wildeman, Adriaan C.I.T.L Tan, Peter D. Siersema

Table 1s. Patient costs, absenteeism and presenteeism according to the IMTA productivity cost questionnaire, pre and post bowel preparation and colonoscopy.

	**1L PEG+Asc (n=238, ITT)** Missing n=14 (5.9%)			**2L PEG+Asc (n=227, ITT)** Missing n=22 (9.6%)		
	**Pre**	**Post**	**p**	**Pre**	**Post**	**p**
**Paid job**, n (%)	121 (54.0)			107 (51.7)		
**Amount of work per week**, mean (SD)		n/a			n/a	
Hours	31.8 (11.7)			32.6 (11.4)		
Days	4.2 (1.1)			4.3 (1.1)		
**Absenteeism in past 4 weeks**						
n (%)	15 (12.4)	16 (7.3)	0.688	22 (20.6)	21 (10.3)	0.070
Number of days, median (IQR)	5 (2-16)	9 (3.3-20)		5 (3.8-9)	7 (4-18)	
Number of working hours, median (IQR)	40 (16-80)	38 (18-140)		40 (22.2-53.8)	42.9 (23.4-124)	
Costs in euros, median (IQR)	1390 (556-2780)	1320 (626-4865)		1390 (770-1870)	1489 (813-4309)	
**Presenteeism**						
n (%)		26 (11.9)	1.0		26 (12.7)	1.0
Number of days, median (IQR)		6 (3-10.5)			(3-10)	
Working efficacy 0-10		5 (4-8)			6 (4-7)	
Number of working hours with reduced efficacy, median (IQR)		-18.4 (-34.4, -6)			-18.1 (-29, -8.7)	
Costs in euros, median (IQR)		-639 (-1193, -209)			628 (-1008, -303)	
**Unpaid labor loss**						
n (%)	20 (8.9)	17 (7.8)	0.629	19 (9.2)	19 (9.3)	1.0
Number of days, median (IQR)	8.5 (3.3-18.8)	20 (6.5-24)		10 (4-21)	6 (4-16)	
Number of working hours lost, median (IQR)	33.5 (6.8-100)	40 (9-142)		40 (6-100	16 (8-80)	
Costs in euros, median (IQR)	469 (94.5-1400)	560 (126-1988)		560 (84-1400)	224 (112-1120)	
**Caregiver help**						
n (%)		180 (82.6)			169 (82.8)	0.977
Caregiver absenteeism, n (%)		55 (30.6)			56 (33.1)	0.721
**Self-reported costs**						
None, n (%)		144 (61.2)			126 (55.5)	
Medication, n (%)		8 (3.4)			6 (2.6)	
Amount, mean (SD)		*13 (27.65)*			*18 (21.75)*	
OTC medication, n (%)		3 (1.3)			5 (2.2)	
Amount, mean (SD)		*5.67 (1.16)*			*13.6 (12.97)*	
Travel costs, n (%)		64 (27.0)			63 (27.8)	
Amount, mean (SD)		*15 (12.51)*			*12.92 (11.27)*	
Other, n(%)		16 (6.8)			22 (9.6)	
Amount, median (IQR)		*6 (1.5-36.3)*			*10 (5-15)*	
**Total self-reported costs**, median (IQR)		10 (5-25)			10 (5.9-20)	0.951
**Sum costs of absenteeism, presenteeism, unpaid labor loss, and self-reported costs**, median (IQR)		17 (9-65.6)			15 (7-39)	0.585

Table 2s. Results of the Mayo Florida Bowel preparation tolerability questionnaire

	**1L PEG+Asc (n=238, ITT)** missing n=19 (8.4)	**2L PEG+Asc (n=229, ITT)** missing n=24 (10.5)	**p-value**
**Number of bowel movements in week prior to prep**, n (%)			0.722
3 or less/week	18 (8.2)	20 (8.7)	
4-8/week	129 (58.9)	124 (60.5)	
9 or more/week	72 (32.9)	61 (26.6)	
**Laxative left after drinking to best effort**, n (%)			0.341
Less than 25%	218 (99.5)	202 (98.5)	
25% - 50% left	1 (0.5)	1 (0.5)	
50% - 75% left	0	2 (1.0)	
75% or more left	0	0	
**Tolerability**, n (%)			0.219
Easy	113 (51.6)	115 (57.1)	
Acceptable	63 (28.8)	63 (30.7)	
Somewhat difficult	27 (12.3)	15 (7.3)	
Very difficult	16 (7.3)	10 (4.9)	
**Willingness-to-repeat this prep**, n (%)			**0.044**
not willing at all	17 (7.8)	16 (7.8)	
somewhat willing	71 (32.4)	90 (43.9)	
mostly willing	131 (59.8)	99 (48.3)	
**In case of experienced difficulties in tolerability, do you think it was due to your current health issues?** n, (%)			0.550<br>0.304 for trend
Yes	6 (2.7)	2 (1.0)	
No	52 (23.7)	46 (22.4)	
N/A (no difficulties)	117 (53.4)	111 (54.1)	
N/A (no health issues)	44 (20.1)	46 (22.4)	
**Symptoms**, n (%)			
Bad taste			
None	65 (29.7)	85 (41.5)	**0.007**
Mild	92 (42.0)	79 (38.5)	
Moderate	4 (18.7)	35 (17.1)	
Severe	21 (9.6)	6 (2.6)	
Feeling full			
None	63 (28.8)	42 (20.5)	0.166
Mild	87 (39.7)	81 (39.5)	
Moderate	55 (25.1)	65 (31.7)	
Severe	14 (6.4)	17 (8.8)	
Lack of sleep			
None	110 (50.2)	80 (39.0)	0.091
Mild	59 (26.9)	61 (29.8)	
Moderate	35 (16.0)	41 (20.0)	
Severe	15 (6.8)	23 (11.2)	
Nausea/vomiting			
None	141 (64.4)	163 (79.5)	**0.006**

Mild	51 (23.3)	30 (14.6)	
Moderate	15 (6.8)	7 (3.1)	
Severe	12 (5.5)	5 (2.4)	
Bloating			
None	67 (30.6)	64 (31.2)	0.749
Mild	99 (45.2)	87 (42.4)	
Moderate	44 (20.1)	41 (20.0)	
Severe	9 (4.1)	13 (6.3)	
Abdominal pain/cramps			
None	104 (47.5)	98 (47.8)	0.960
Mild	84 (38.4)	75 (36.6)	
Moderate	25 (11.4)	25 (12.2)	
Severe	6 (2.7)	7 (3.4)	
Headache			
None	155 (70.8)	149 (72.7)	0.212
Mild	42 (19.2)	43 (21.0)	
Moderate	15 (6.8)	12 (5.9)	
Severe	7 (3.2)	1 (0.5)	
**Symptom score***, mean (SD)	5.6 (3.8)	5.5 (3.4)	0.743
**What is the tolerability of the laxative used compared to the previous experience?** N (%)	N=105	N=98	**<0.001**
Worse	12 (5.5)	13 (6.3)	
About the same	34 (15.5)	66 (32.2)	
Better	59 (26.9)	19 (9.3)	
N/A (first colonoscopy)	114 (52.1)	107 (52.2)	

Table 3s. Factors associated with willingness to repeat in univariate and multivariate logistic regression. OR, Odds ratio; CI, confidence interval; Mayo bowel preparation tolerability questionnaire;

**Predictor**	**OR univariate (95% CI)**	**p-value**	**OR multivariate (95% CI)**	**p-value**	**OR final model (95% CI)**	**p-value**
2L PEG+Asc compared to 1L PEG+Asc	0.622 (0.432-0.896)	**0.011**	0.367 (0.212-0.637)	**<0.001**	0.400(0.239-0.669)	<0.001
Age	1.008 (0.992-1.024)	0.343				
Sex female	0.488 (0.336-0.709)	**<0.001**	1.094 (0.610-1.960)	0.763		
No prior experience	0.822 (0.572-1.183)	0.292				
Colonoscopy indication<br>*Screening*<br>*Surveillance*<br>*Diagnostic*	 <br>Reference<br>1.301 (0.834-2.028)<br>0.838 (0.537-1.305)	**0.161**	 <br>Reference<br>1.544 (0.610-1.960)<br>1.724 (0.835-3.560)	0.189		
Education level<br>*None*<br>*Secondary school*<br>*University*	 Reference<br>0.661 (0.182-2.401)<br>0.724 (0.197-2.656)	0.765				
Married/living together	1.858 (1.118-3.088)	**0.017**	1.598 (0.787-3.245)	0.194		
No paid occupation	0.752 (0.515-1.100)	**0.142**	0.691 (0.401-1.192)	0.184		
No excellent prep	0.928 (0.628-1.371)	0.707				
Gloucester comfort scale score<br>1<br>2<br>3/4/5	 <br><br>Reference<br>0.829 (0.546-1.260)<br>0.577 (0.359-0.929)	**0.077**	 <br><br>Reference<br>0.959 (0.520-1.767)<br>0.837 (0.393-1.786)	0.893		
Polyp detected	1.355 (0.936-1.962)	**0.108**	0.975(0.540-1.760)	0.933		
No absenteeism	1.616 (0.818-3.192)	**0.167**	1.370 (0.457-4.108)	0.574		
No presenteeism	1.441 (0.805-2.579)	0.219				
Tolerability (MBTQ)<br>*good*<br>*fair*<br>*difficult*	 <br>Reference<br>0.178 (0.111-0.286)<br>0.029 (0.012-0.071)	**<0.001**	 <br>Reference<br>0.208 (0.115-0.375)<br>0.054 (0.019-0.155)	**<0.001**	 <br>Reference<br>0.225 (0.130-0.389)<br>0.053 (0.020-0.143)	<0.001
Symptom score	0.726 (0.673-0.783)	**<0.001**	0.826 (0.744-0.918)	**<0.001**	0.852 (0.779-0.932)	<0.001
Total endoscopy satisfaction score	1.067 (1.039-1.095)	**<0.001**	1.054 (1.015-1.095)	**0.006**	1.042 (1.008-1.076)	0.015
EQ VAS	1.031 (1.016-1.047)	**<0.001**	1.004 (0.977-1.031)	0.770		
EQ Index	1.040 (1.021-1.059)	**<0.001**	1.009 (0.989-1.029)	0.404		

Table 4s. EQ-5D-5L results before (pre) and after (post) bowel preparation.

**Category** median (IQR)	**1L PEG+Asc (n=238, ITT)**				**2L PEG+Asc (n=227, ITT)**				**P-value of change**
	**Pre**	**Post**	**Change** mean (SD)	P pre vs post	**Pre**	**Post**	**Change** mean (SD)	P pre vs post	
Mobility	1 (1-1)	1 (1-1)	0.01 (0.59)	0.969	1 (1-1)	1 (1-1)	0.08 (0.42)	**0.008**	0.171
Selfcare	1 (1-1)	1 (1-1)	-0.02 (0.21)	0.096	1 (1-1)	1 (1-1)	0.01 (0.28)	1.0	0.233
Activity	1 (1-1)	1 (1-1)	-0.08 (0.71)	0.105	1 (1-1)	1 (1-1)	-0.01 (0.53)	0.894	0.257
Pain	1 (1-2)	1 (1-2)	0.17 (0.79)	**0.002**	1 (1-2)	1 (1-2)	0.08 (0.60)	0.075	0.119
Anxiety	1 (1-1)	1 (1-1)	0.14 (0.59)	**0.001**	1 (1-1)	1 (1-1)	0.06 (0.52)	0.067	0.198
VAS, mean (SD)	80.41 (14.0)	79.95 (13.2)	-0.29 (12.6)	**<0.001**	81.85 (13.4)	81.0 (13.2)	-0.60 (9.8)	**<0.001**	0.963
EQ Index	0.89 (0.82-1.0)	1 (0.85-1.0)	-0.03 (0.13)	**<0.001**	0.92 (0.85-1.0)	1 (0.85-1.0)	-0.02 (0.10)	**<0.001**	0.440

Table 5s. Results of the Dutch gastrointestinal endoscopy satisfaction questionnaire. Higher scores indicate higher satisfaction, 0=worst and 100=best.

mean scores (SD)	**Complete study population, n=42** missing n=77	**1L PEG+Asc (n=238, ITT)** Missing, n=39 (16.4%)	**2L PEG+Asc (n=227, ITT)** Missing, n=38 (16.6%)	**p-value between study arms**
**Skills and satisfaction**	92.4 (8.8)	92.4 (8.5)	92.5 (9.0)	0.849
**Information before endoscopy**	85.4 (10.1)	85.3 (10.3)	85.4 (10.0)	0.941
**Pain and discomfort**	86.3 (12.6)	86.4 (12.6)	86.1 (12.7)	0.912
**Information after endoscopy**	84.0 (10.3)	84.0 (10.7)	84.0 (10.0)	0.828
**Hospital**	90.5 (11.5)	89.4 (12.3)	91.6 (10.5)	0.053
**Total**	85.1 (8.3)	84.9 (8.5)	85.2 (8.2)	0.405
